# The complete mitochondrial genome of *Epicauta sibirica* Pallas, 1773 (Coleoptera: Meloidae)

**DOI:** 10.1080/23802359.2024.2418140

**Published:** 2024-11-11

**Authors:** Nan Yang, Suhua Zhang, Nashen Jin, Ning Wang, Zilong He, Yufang Xu, Kejian Lin

**Affiliations:** a College of Prataculture, Qingdao Agricultural University, Qingdao, China; b Hohhot Forestry and Grassland Conservation Center, Hohhot, China; c Hulun Buir Forestry Group Co., Ltd, Hulun Buir, China; d Institute of Grassland Research, Chinese Academy of Agricultural Sciences, Hohhot, China; e Xilin Gol League Grassland Workstation, Inner Mongolia, China

**Keywords:** *Epicauta sibirica*, mitochondrial genome, phylogeny, genome map

## Abstract

*Epicauta sibirica* is both an important pest and an important economic insect. The mitogenome is 15,717 bp in length and consists of 13 protein-coding genes, 2 rRNA genes, 22 tRNA genes, and a control region. The nucleotide composition of *E. sibirica* is 37.0% of A, 18.3% of C, 11.3% of G, and 33.4% of T. The genus *Epicauta* was detected as a monophyletic group and more closely related to the genus *Lytta* than other genera within Meloidae. This study supplemented additional genetic data for future research on the phylogeny and evolution of Meloidae species.

## 1. Introduction

The genus *Epicauta* Dejean, 1834 is widespread in the Palearctic region east of the Altai Mountains and in the transition zone between Palearctic and Oriental regions (Zhang et al. 2019). It’s one of the largest genera of Meloidae, possessing approximately 400 species (Liu et al. 2016; Bologna et al. 2008; Campos-Soldini 2022).

**Figure 1.**
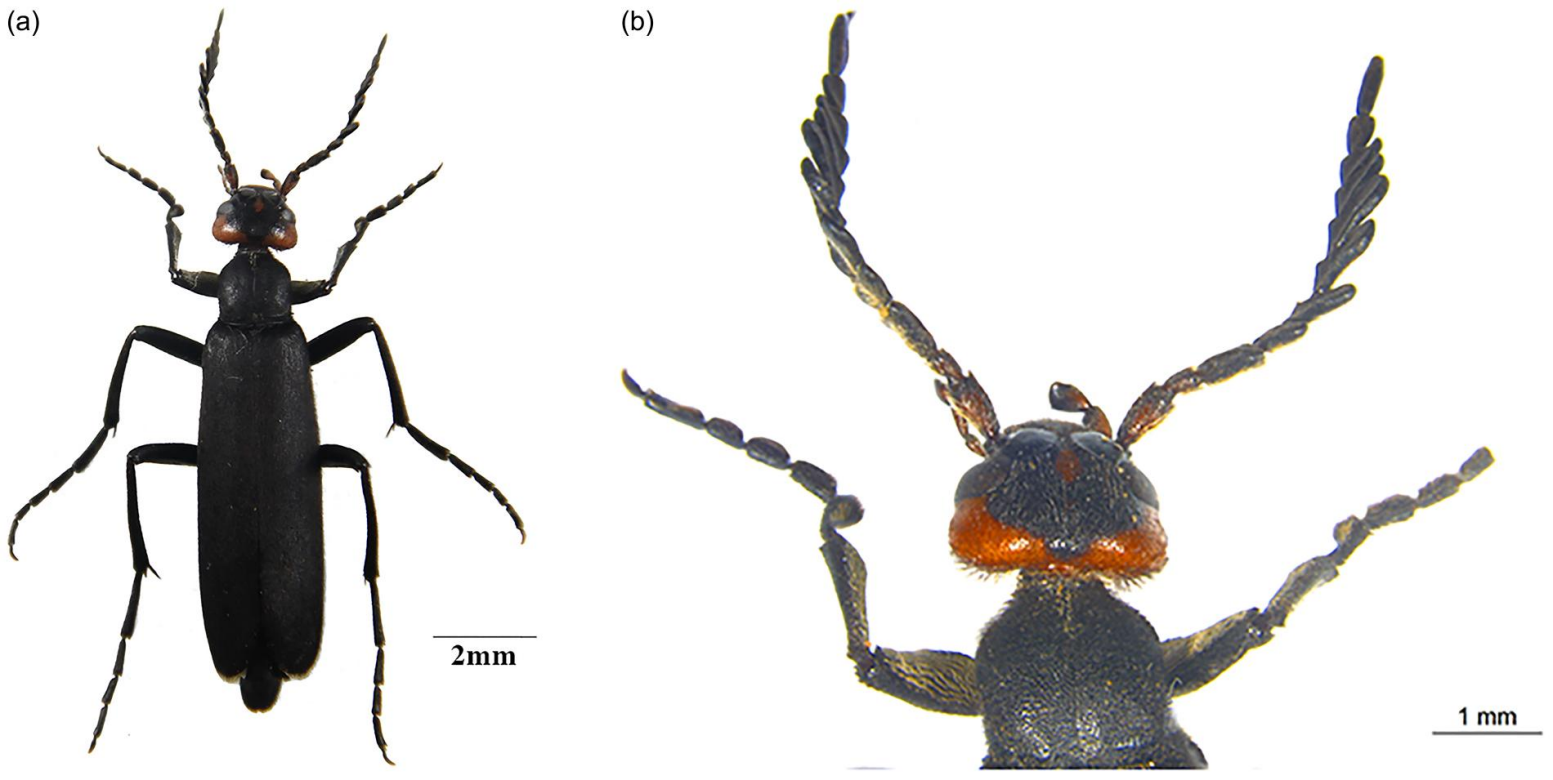
(a) Full-body photograph of *Epicauta sibirica.* The insect was collected by Guangming Liu from Sunite Right Banner(42°47′3″N,112°40′21″E), Inner Mongolia, China. The images were taken by Nan Yang, using microscope Camera Equipment 0.5X MC120HD and (b) A photograph of localized features of *Epicauta sibirica* was taken by Nan Yang with Microscope: Camera Equipment 0.5X MC120HD, Eyepieces*10 and Objectives*20, focus stacked with camera software LAS Multi Focus.

Although *E. sibirica* Pallas, 1773 is an important crop pest, in recent years it has become a research hotspot in medical drug development and plant protection (Zhang et al. 2019). Known as the blister beetle, Meloidae secretes sesquiterpene substance, cantharidin (C_10_H_12_O_4_) from its legs and antennae when stimulated, and direct contact can cause blisters on human skin (Fratini et al. 2021). Although it is nephrotoxic and highly toxic for internal use in traditional Chinese medicine, *E. sibirica* was used as a topical drug to relieve skin itching, and in recent years, cantharidin has been studied more in terms of its anticancer potential (Naz et al. 2020). The larvae *E. sibirica* is commensal parasitism in locust eggs, and it is widely used for biological control due to its insecticidal activity (Tan 1958; Tian et al. 2021). The distal segment of the antenna is slightly longer than the anterior segment (Yang 2007). In male *E. sibirica*, antennal segments 4–9 are subpectinate, whereas in females all antennal segments are filiform (Pan et al. 2011) (Figure 1). There are coarse and dense punctations on the head of *E. sibirica*. Many scholars have studied the biological behavior ability of Meloinae, and current research in the Meloinae focuses on species identification through morphological differences and genetic variability, phylogenetic analyses, and the geographical distribution of species (Campos-Soldini 2022). There are few studies on *E. sibirica*, especially on the genomic level. Until now, there are only 13 genome sequences of *E. sibirica* released on NCBI (Table S2). Our study will publish the first complete mitochondrial genome sequence of *E. sibirica*, providing more available data for further phylogenetic studies.

## 2. Materials and Methods

### 2.1. Materials

The adult *Epicauta sibirica* specimens, collected from Sunite Right Banner, Inner Mongolia, China (42°47′3″N, 112°40′21″E) on 12th July 2021 by Guangming Liu, were preserved in the Entomological Museum of the Institute of Grassland Research, Chinese Academy of Agricultural Sciences (No. IGR600072, Ning Wang: wangningis@163.com). After we took pictures of the original specimens, Professor Pan Zhao identified them as *E. sibirica*. (Figure 1). The Xilinhot Forestry and Grassland Bureau approved the sample collection. The Administration Committee of Experimental Animals of Inner Mongolia Province and the Ethics Committee of the Chinese Academy of Agricultural Sciences authorized all animal operations and experimental courses.

### 2.2. Methods

**Figure 2.**
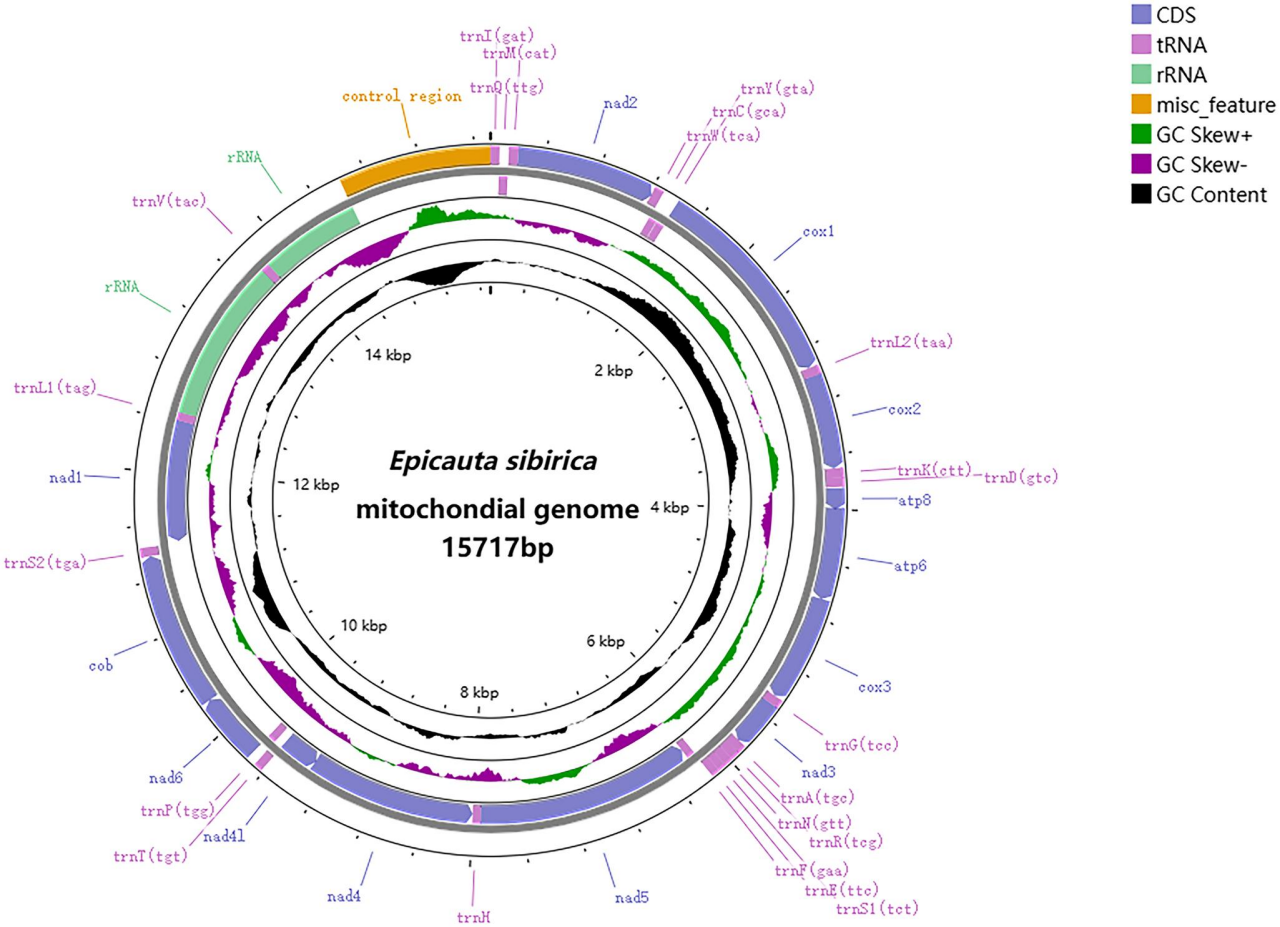
Genome map of *Epicauta sibirica*. Circular maps were drawn with GCView. The outermost circle indicates the arrangements of genes: outer genes from the forward strand, and inner genes from the reverse strand. GC-skew was plotted (positive skew in green, negative skew in purple) as the deviation from the average GC-skew of the entire sequence.

DNA materials were extracted from the whole body of a single adult using the TrueLib DNA Library Rapid Prep Kit (ExCell, Jiangsu, China). The DNA sample was sent to Beijing Biomarker Biotechnology Co. to build library for sequencing on the Illumina Novaseq 6000 platform. After quality checking and assembling with MitoZ v2.3 (Meng et al. 2019), the filtered reads (4.2GB) were examined for an average sequence coverage depth of 1733× (Figure S1). We firstly checked whether the assembled fragment is looped before conducting annotation on the MITOS web server (Bernt et al. 2013), and then the annotation was checked manually on MEGA v7.0 (Kumar et al. 2016) by aligning genes with other published mitochondrial genes of homologous sequences of *E. sibirica*. The mitochondrial genome circle was mapped and landscaped using CGView (https://cgview.ca) (Figure 2).

**Figure 3.**
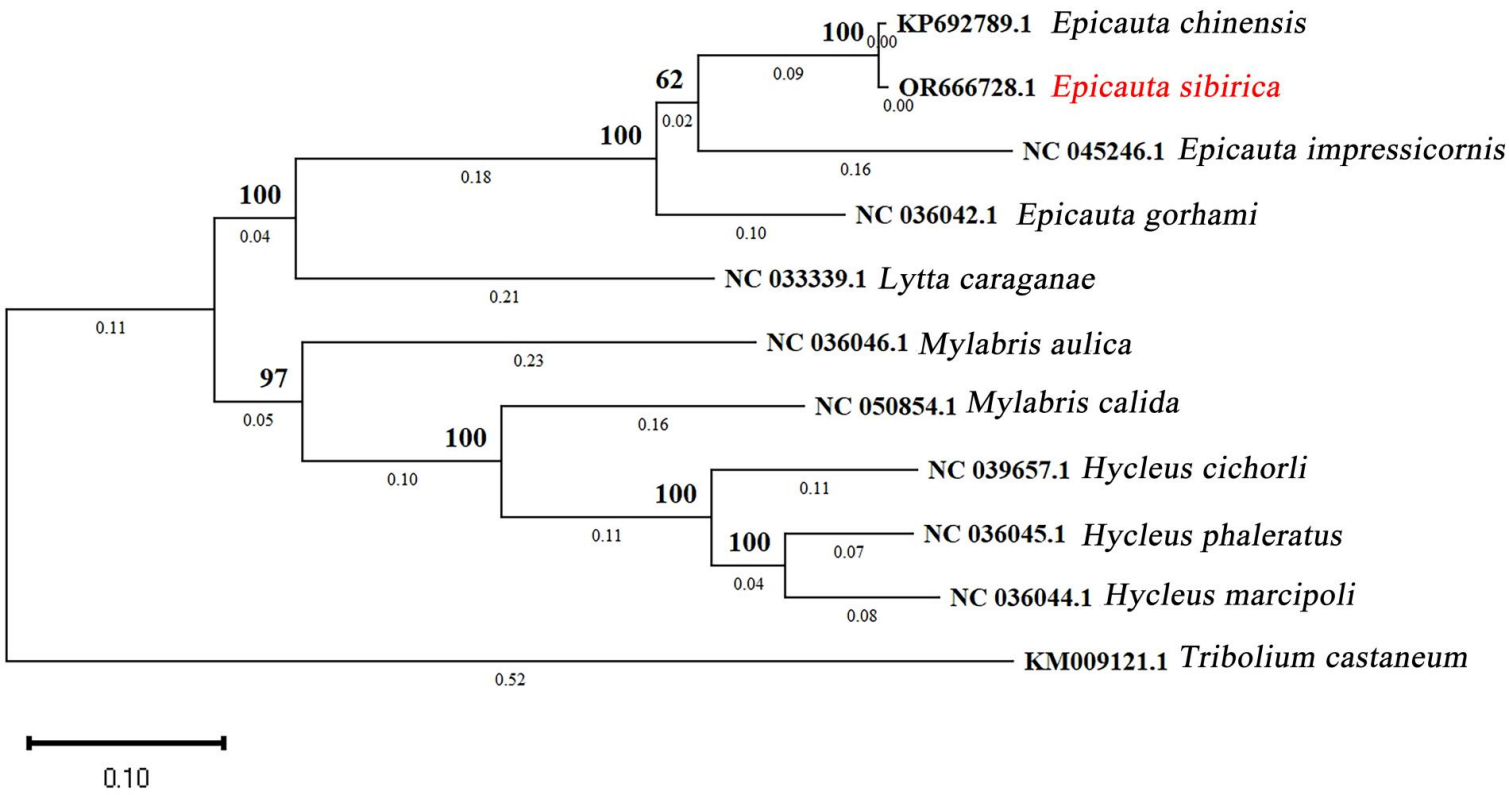
The phylogenetic tree was constructed by 11 species from Coleoptera based on their whole mitochondrial genome by maximum likelihood (ML) method. GeneBank accession numbers of all sequences have displayed in the figure with their corresponding names. Our published data was marked in red. *E. chinensis* (KP692789.1) (Du et al. 2016); *E. impressicornis* (NC_045246.1) (Liu et al. 2020); *E. gorhami* (NC_036042.1) (Du et al. 2017); *M. calida* (NC_050854.1) (Jiang et al. 2020); *M. aulica* (NC_036046.1) (Du et al. 2017); *H. cichorii* (NC_039657.1) (Wu et al. 2018); *H. phaleratus* (NC_036045.1) (Wu et al. 2018); *H. marcipoli* (NC_036044.1) (Du et al. 2017); *L. caraganae* (NC_033339.1) (Unpublished); *Tribolium castaneum (KM009121.1)* (Liu et al. 2016); *Epicauta sibirica* (OR666728.1) (this study).

To test the reliability of sequence assembling and explore the taxonomic status of *E. sibirica*, phylogenetic analyses of Meloinae were performed using the protein-coding genes and rRNA genes of mitogenomes of *E. sibirica* and other ten species data from NCBI, including one species of the genus *Lytta* Fabricius, 1775 (*Lytta caraganae* Pallas, 1781), two of the genus *Mylabris* Fabricius, 1775 (*Mylabris calida* Pallas, 1782; *Mylabris aulica* Ménétriés, 1832), three of the genus *Hycleus* Latreille, 1817 (*Hycleus cichorii* Linnaeus, 1758; *Hycleus phaleratus* Pallas, 1781; *Hycleus. Marcipoli* Pan & Bologna, 2014), and three of the genus *Epicauta* (*Epicauta. chinensis* Laport, 1840; *Epicauta impressicornis* Pic, 1913; *Epicauta gorhami* Marseul, 1873). *Tribolium castaneum* Herbst (Coleoptera: Tenebrionidae) was served as the out-group. After all mitochondrial gene sequences aligned with ClustalW on MEGA v7.0, yielding a dataset of 14,670 bp total in length. The maximum likelihood inference of 11 mitochondrial nucleotide sequences was performed on MEGA v10.0 (Kumar et al. 2018), with a bootstrap value of 1000. The molecular evolution model was the best GTR+I + G model. The phylogenetic tree was landscaped on iTOL (http://itol.embl.de) (Figure 3). Considering the close relationship between *E. sibirica* and *E. chinensis* in former studies (Liu et al. 2016), we also made a genetic distance comparison. The table of pairwise distance was exported by MEGA v10.0 (Table S1).

## 3. Result

The mitochondrial genome is 15717 bp in total length (GenBank accession number: OR666728.1) and consists of 13 protein-coding genes (ND1–ND6, ND4L, COX1–COX3, ATP6, ATP8, Cytb), 22 tRNAs, 2 rRNAs and a control region, of which protein-coding genes account for 71% of the total genome, tRNAs for 9%, and rRNAs for 13%. The gene structure and arrangement are similar to that of other *Epicauta* (Han et al. 2020; Jie et al. 2016; Du et al. 2016; Liu et al. 2020). The total nucleotide composition of the genome is 37.0% A, 18.3% C, 11.3% G, and 33.4% T. The AT content (70.4%) is substantially higher than the GC content (29.6%) like those of other species of Meloidae. (Song et al. 2022; Han et al. 2020; Jie et al. 2016; Wu et al. 2018). For the PCGs, four genes (COX1, ND5, ND6, ND1) use ATT as the start codon, three genes (ND2, COX2, ATP8) take the start codon ATA, five genes (ATP6, COX3, ND4, Cytb, ND4L) take the start codon ATG, and the start codon of the ND3 is ATC. The conventional stop codons are used by all PCGs (TAG for ND1 and ND3, TAA for other genes). The length of tRNAs ranges from 57 to 70 bp and has a cloverleaf shape. In the phylogenetic results, the pairwise distance of mitochondrial genome between *E. sibirica* and *E. chinensis* is 0.7% (Table S1). A monophyletic *Epicauta* species was sister to *L. caraganae* (Figure 3).

## 4. Discussion and conclusion

In the phylogenetic results, there is a short genetic distance between *E. sibirica* and *E. chinensis*. Liu suggested that *E. chinensis* is a junior synonym of *E. sibirica* (Liu et al. 2016). However, confirming the relationship between *E. sibirica* and *E. chinensis* requires more rigorous proof such as hybridization experiments between the two species. The evolutionary relationships among genera of Meloidae are consistent with previous studies (Song et al. 2022; Liu et al. 2020). Moreover, our research provides valuable information about the whole mitogenome of *E. sibirica*, which will support future studies on phylogeny, species identification, and the development of molecular markers. The phylogenetic analysis suggested to differentiate *E. sibirica* using mitochondrial gene sequences.

## Supplementary Material

Pairwise distance.xlsx

The 13 accession numbers for E sibirica currently published on NCBI.xlsx

## Data Availability

The genome sequence data are openly available in GenBank of NCBI at https://www.ncbi.nlm.nih.gov/ under the accession No. OR666728.1. The associated BioProject, SRA, and Bio-Sample numbers are PRJNA779250, SRR16898033, and SAMN23019658, respectively.
